# Mass Spectrometry Reveals α-2-HS-Glycoprotein as a Key Early Extracellular Matrix Protein for Conjunctival Cells

**DOI:** 10.1167/iovs.61.3.44

**Published:** 2020-03-30

**Authors:** Aruni K. Makuloluwa, Rosalind M. K. Stewart, Stephen B. Kaye, Rachel L. Williams, Kevin J. Hamill

**Affiliations:** 1 Department of Eye and Vision Science, University of Liverpool, Liverpool, United Kingdom; 2 St. Paul's Eye Unit, Royal Liverpool University Hospital, Liverpool, United Kingdom; 3 St. James’ University Hospital, Leeds, United Kingdom

**Keywords:** ocular surface, conjunctiva, extracellular matrix, tissue engineering, mass spectrometry

## Abstract

**Purpose:**

To determine the composition of extracellular matrix (ECM) proteins secreted by a conjunctival epithelial cell line and to identify components that aid conjunctival epithelial cell culture.

**Methods:**

Human conjunctival epithelial cell line (HCjE-Gi) cells were cultured in serum-free media and their ECM isolated using ammonium hydroxide. Growth characteristics were evaluated for fresh HCjE-Gi cells plated onto ECMs obtained from 3- to 28-day cell cultures. Mass spectrometry was used to characterize the ECM composition over 42 culture days. Cell adhesion and growth on pre-adsorbed fibronectin and α-2-HS-glycoprotein (α-2-HS-GP) were investigated.

**Results:**

Day 3 ECM provided the best substrate for cell growth compared to ECM obtained from 5- to 28-day cell cultures. Mass spectrometry identified a predominantly laminin 332 matrix throughout the time course, with progressive changes to matrix composition over time: proportional decreases in matrix-bound growth factors and increases in proteases. Fibronectin and α-2-HS-GP were 5- and 200-fold enriched as a proportion of the early ECM relative to the late ECM, respectively. Experiments on these proteins in isolation demonstrated that fibronectin supported rapid cell adhesion, whereas fibronectin and α-2-HS-GP both supported enhanced cell growth compared to tissue culture polystyrene.

**Conclusions:**

These data reveal α-2-HS-GP as a candidate protein to enhance the growth of conjunctival epithelial cells and raise the possibility of exploiting these findings for targeted improvement to synthetic tissue engineered conjunctival substrates.

The conjunctiva is an integral component of the ocular surface system, protecting the eyes from external insults by acting as a physical and an immunological barrier.^1^^,^^2^ It does so through forming a stratified non-keratinizing epithelium comprised of epithelial cells and mucosecretory goblet cells adhered to a basement membrane.^3^ The conjunctiva contains its own pool of stem cells for epithelial and goblet cell renewal.^2^ The epithelial and goblet cells produce mucins, which act as a second barrier layer and also prevent desiccation of the epithelium.^2^^,^^3^ Despite the existence of multiple defense mechanisms, the conjunctiva is prone to injury and entry of pathogens or damage as a result of systemic autoimmune diseases.^4^^,^^5^ Subsequent scarring may lead to anatomical and functional impairment. In severe cases, the limbus and cornea may also be impaired leading to loss of vision.^6^ Importantly, surgical procedures such as limbal and corneal transplantation fail without prior restoration of the function of the conjunctival epithelia.^3^^,^^7^ The ideal conjunctival repair is an autograft graft.^8^ Diseases, however, often involve both eyes or there is insufficient uninvolved tissue in the fellow eye for this to be available.^9^ Although allogeneic grafts may be an option, this approach carries the risks of graft rejection, microbial disease transmission, and immunosuppression, and there are limitations to tissue availability.^10^ An attractive alternative, therefore, is the development of a functional ex vivo tissue construct that can be transplanted to restore the anatomical structure and functions of the original tissue.

The success of an ex vivo expanded tissue construct depends on three factors: the source of cells, the presence of stem cells, the carrier substrate, and the presence of growth factors (GFs).^11^ The substrate provides the initial support for the cultured cells and the appropriate physical and biological cues for growth of cells.^12^ Moreover, the physical properties of the substrate facilitates the delivery of cultured cells to the site of interest and provides a three-dimensional scaffold for the formation of new tissue.^13^ A large number of studies have been carried out to investigate various biological and synthetic substrates in the search for an optimal substrate for the ex vivo culture of conjunctival epithelial cells and, although substantive advancements have been made, significant technical challenges still remain.

Test substrates fall into two classes: (1) biological substrates, such as amniotic membrane and nasal and oral mucous membranes, and (2) synthetic substrates, such as poly(ε-caprolactone) and poly(lactic-*co*-glycolic acid).^10^^,^^14^^–^^17^ Biological substrates are attractive cell carrier options, as they are generally biodegradable and contain biologically active domains that support various cellular functions.^18^ However, biological substrates have inferior mechanical properties, which make them more difficult to handle clinically. Additionally, they may be limited in supply, they may elicit an immunological reaction in vivo, and, when taken from a different body location, may retain signals that drive differentiation of cells down an inappropriate lineage.^18^ In contrast, synthetic substrates have a known, tunable, and consistent composition that can be modified to suit requirements and benefit from constant ready supply. However, there are clear disadvantages in terms of lack of structural similarity to in vivo cellular microenvironments and non-biologically accurate presentation of the signaling cues.^19^

The extracellular matrix (ECM) regulates the growth and differentiation of cells, thus providing specific biological and physical cues.^12^^,^^20^ The vast majority of studies driving development of synthetic carriers have investigated a relatively narrow range of candidate biologically active ECM molecules and their derived peptides.^3^^,^^18^^,^^21^^,^^22^ Here, we have approached the problem from a different angle. We hypothesized that the best substrate for growing conjunctival epithelial cells is likely to be the substrate laid down by healthy cells themselves. Identifying biologically active components within a healthy culture of conjunctival cells could provide a valuable first step toward an improved synthetic substrate for cell and tissue transplantation purposes. To investigate this, we performed a hypothesis-independent, mass-spectrometry-based characterization of the composition of the native ECM of conjunctival cells in culture and used those data to identify specific components that aid conjunctival cell culture.

## Methods

### HCjE-Gi Cell Culture

Cells from the immortalized human conjunctival epithelial cell line (HCjE-Gi; kindly donated by Gipson Laboratory at Schepens Eye Research Institute-Massachusetts Eye and Ear, Harvard Medical School, Boston, MA, USA) were cultured and maintained according to published protocol.^23^ Briefly, the cells were cultured in keratinocyte serum-free (KSF) media (Life Technologies, Carlsbad, CA, USA) containing 25-µg/mL bovine pituitary extract and 0.2-ng/mL human recombinant epidermal growth factor 1-53 (supplied with media), 0.4-mM calcium chloride (Sigma-Aldrich, St. Louis, MO, USA), and 1× penicillin–streptomycin (Sigma-Aldrich) in a humidifying incubator at 37°C with 5% carbon dioxide. The cells were maintained in 75-cm^2^ flasks (Greiner AG, Kremsmünster, Austria), and fresh media were supplied three times a week. Cells were used in experiments with an initial seeding density of 1 × 10^4^ cells/cm^2^ unless specified otherwise. At this seeding density, cells progressed from approximately 10% to 90% confluent through the first 7 days after seeding (Supplementary Figs. S1A, S1B)

### Isolation of the ECM

Ammonium hydroxide (NH_4_OH) was used to remove the cells and isolate the underlying ECM.^24^^,^^25^ Culture media were removed, and the cells were washed three times with PBS (Thermo Fisher Scientific Oxoid Ltd., Basingstoke, United Kingdom). The cells were then incubated in 1% NH_4_OH solution (Fisher Scientific, Hampton, NH, USA) for 5 minutes at 37°C. The NH_4_OH solution was removed, and the wells were washed vigorously with PBS three times to remove cellular debris, leaving only the deposited ECM. For cell behavioral analyses, fresh HCjE-Gi cells were plated directly onto the prepared cell-derived ECMs. For mass spectrometry, cell-derived ECMs were solubilized from the dishes.

### Comparison of HCjE-Gi Cell Response to Cell-Deposited ECMs

HCjE-Gi cells were cultured in 48-well plates (Greiner). On culture days 3, 5, 7, 14, 21, and 28, the cell-secreted ECM was isolated. Following thorough PBS washes, fresh HCjE-Gi cells were seeded onto the cell-deposited ECMs at a density of 1 × 10^4^ cells/cm^2^ in KSF culture media. Tissue culture polystyrene (TCP) incubated with 1% NH_4_OH solution and then thoroughly washed with sterile PBS was used as the control substrate. We then assessed the cell numbers on the different culture conditions on culture days 1, 3, 5, and 7 after seeding. The cells were fixed with 100% ice-cold methanol for 2 minutes, air dried, and stained with 4′,6-diamidino-2-phenylindole (DAPI) (Life Technologies; 5 mg/mL diluted 1:30,000 in PBS) for 5 minutes at room temperature (RT). Images were acquired with a 10× objective from five fixed positions of each well, and the number of nuclei per view was determined. The number of nuclei per view on cell-secreted ECM was divided by the number of nuclei of the corresponding view on TCP to obtain ECM:TCP cell number ratios. All experiments were carried out three times, with three technical repeats per independent experiment.

### Identification and Quantification of HCjE-Gi Cell-Secreted ECM Proteins by Mass Spectrometry

HCjE-Gi cells were cultured in 48-well plates and maintained in KSF media for up to 6 weeks. The ECM was isolated using 1% NH_4_OH from cell cultures on days 1, 7, 14, 28, and 42 after seeding. The samples were dried and stored at –20°C until all of the samples were collected. The samples were then thawed, and RapiGest SF (Waters Corporation, Milford, MA, USA), an anionic surfactant, was used to solubilize the ECM directly on the dishes for subsequent in-solution tryptic digestion. RapiGest SF has been demonstrated to be highly effective for ECM protein solubilization.^26^^,^^27^ First, 285 µL of 0.052% (w/v) RapiGest SF surfactant reconstituted in ammonium bicarbonate (NH_4_HCO_3_) (Sigma-Aldrich) was added to each sample well (final concentration 0.05%). The samples were incubated with the RapiGest SF solution for 30 minutes at RT on a plate shaker (VXR basic Vibrax, IKA Werke GmbH, Staufen, Germany) and then for 10 minutes at 80°C. The samples were cooled to RT and transferred into 1.7-mL low-binding tubes (Corning Inc., Corning, NY, USA). To ensure maximum ECM recovery, each well was rinsed with a further 100 µL of RapiGest SF, and this was transferred to the same low-binding tubes and the samples gently vortexed. Half of the protein extract (192.5 µL) was aspirated into new low-binding tubes, and 2.5 µL of 60-mM dithiothreitol (Melford Biolaboratories Ltd, Ipswich, United Kingdom) reconstituted in 25-mM NH_4_HCO_3_ (final concentration of 0.75 mM) was added. The samples were vortexed and incubated at 60°C for 10 minutes. Next, 2.5 µL of 180-mM iodoacetamide (Sigma) reconstituted in 25-mM NH_4_HCO_3_ (final concentration of 2.25 mM) was added and incubated for 30 minutes at RT in the dark. Finally, 100-ng/µL trypsin (Trypsin Gold Mass Spectrometry Grade; Promega, Fitchburg, WI, USA) was made by diluting 200-ng/µL stock solution in 50-mM acetic acid (VWR, Radnor, PA, USA) with an equal volume of 25-mM NH_4_HCO_3_. The samples were incubated at 37°C overnight with 2.5 µL of trypsin.

The following day the samples were mixed with 1 µL of trifluoroacetic acid (Biosolve Chimie, Dieuze, France). The samples were incubated at 37°C for 45 minutes and then centrifuged at 17,200*g* for 30 minutes. The clarified digests were transferred to fresh low-binding tubes and the centrifugation step repeated before transfer to total recovery vials for liquid chromatography tandem mass spectrometry (LC-MS/MS) analysis. Data-dependent LC-MS/MS analyses were conducted on a Q Exactive HF Hybrid Quadrupole-Orbitrap Mass Spectrometer coupled to a Dionex UltiMate 3000 RSLCnano Liquid Chromatograph system (Thermo Fisher Scientific, Waltham, MA, USA). Please refer to Supplementary Methods S1 for further details.

As the total cell number increased over the time course of the study, the raw data with the number of peptides of each protein found in each sample were incorporated into downstream analyses. The raw peptide counts were divided by the total peptide count for each time point to determine the proportion of each protein within a sample.

### Adhesion and Cell Density of HCjE-Gi Cells on Pre-Adsorbed Proteins

Solutions containing 0.5- to 10-µg/mL fibronectin from human plasma (Sigma-Aldrich) and 0.5- to 20-µg/mL α-2-HS-glycoprotein (α-2-HS-GP) from human plasma (Sigma-Aldrich) were prepared in PBS. The 96-well plates (Greiner) were pre-adsorbed with protein solutions. The wells were washed three times with PBS, and HCjE-Gi cells were seeded at a density of 3 × 10^4^ cells/cm^2^ and incubated for 3 hours for adhesion experiments. To investigate the population densities, the cells were cultured for 1, 3, 5, and 7 days with initial seeding densities of 1 × 10^4^ cells/cm^2^. The cells were fixed with 100% ice-cold methanol for 2 minutes, air dried, and stained with DAPI for 5 minutes at RT. HCjE-Gi cells cultured on TCP (0 µg/mL protein) were used as internal calibration control. Images were taken with a 10× objective from five fixed positions of each well, and the number of nuclei per view was determined to calculate the median number of cells per square centimeter in each well. Experiments were carried out three independent times, with sets of three technical repeat wells per experiment.

### Expression of Conjunctival Cell Markers

On culture day 7, the cells were fixed with 10% neutral buffered formalin (Sigma-Aldrich) for 10 minutes at RT, washed three times with PBS, and permeabilized with 0.2% Triton X-100 (Sigma-Aldrich) for 15 minutes at RT. The cells were washed three times in PBS and were incubated with 10% goat serum for 1 hour at RT. The cells were incubated overnight at 4°C with rabbit monoclonal antibodies to keratin 19 (EP15804; Abcam, Cambridge, MA, USA), rabbit polyclonal antibodies to keratin 1 (Abcam), or mouse monoclonal antibodies to keratin 8 and 18 (NCL5D3; Abcam) or to keratin 7 (SC23876; Santa Cruz Biotechnology, Heidelberg, Germany) diluted in 1% BSA. Following three washes with PBS containing Tween (Sigma-Aldrich), the cells were incubated with Alexa Fluor 594 Goat Anti-Rabbit antibodies (final concentration 4 µg/mL; Life Technologies) diluted in 1% BSA for another 1 hour at 37°C and counterstained with DAPI. Images were taken with a 20× objective. Mouse or rabbit immunoglobulins were used instead of the primary antibody as an isotype control to ensure the specificity of the antibodies. Additionally, adult retinal pigment epithelial cell line (ARPE-19),^28^ corneal epithelial cell line (hTCEpi),^29^ or epidermal keratinocyte cell line (HaCaT)^30^ were processed alongside to confirm specificity of the primary antibodies. All images were taken with a Nikon Eclipse Ti-E microscope (Tokyo, Japan). The Image-Based Tool for Counting Nuclei plug-in for Image J software (National Institutes of Health, Bethesda, MD, USA) was used to count cells.

### Statistics

All data were analyzed using Prism 6.1 software (GraphPad, San Diego, CA, USA). One-way ANOVA with Dunnett's multiple comparison test was used to analyze the cell adhesion experiments on pre-adsorbed proteins. Two-way ANOVA with Tukey's multiple comparison test was used to analyze growth experiments across ECM preparations and upon pre-adsorbed proteins, as well as to analyze the peptide abundance of protein categories from ECM days 1 to 42. A type I error rate of 5% was set as the threshold for statistical significance for all experiments.

## Results

### Cell-Derived ECMs from 3-Day HCjE-Gi Cell Cultures Supported Increased Cell Densities Compared to ECM from 5- to 28-Day Cultures

**Figure 1.**
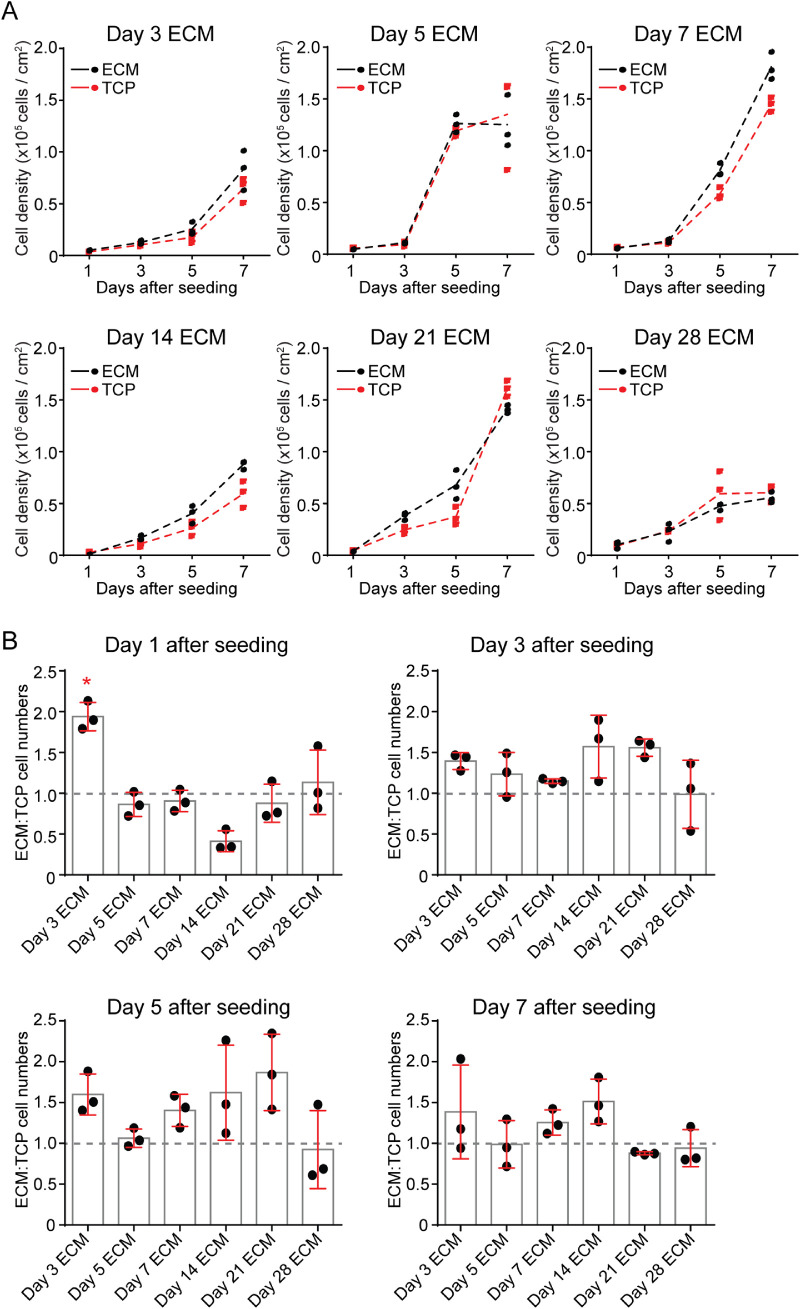
Early ECM is superior to late ECM at supporting HCjE-Gi cell adhesion and expansion. HCjE-Gi cells were cultured for 3, 5, 7, 14, 21, or 28 days to obtain cell-derived ECMs. Fresh HCjE-Gi cells were then plated on top. (**A**) The cell density of HCjE-Gi plated onto cell-derived ECMs (black dots/line) are compared against cells plated in parallel on TCP (red dots/line). Each dot represents one assay with dotted line at mean. (**B**) The ratio of cell density on ECMs relative to TCP internal controls is plotted. Each dot represents one assay, with bars representing mean and standard deviation error bars. The asterisk (*) denotes significant difference (*P* < 0.05) compared with all other ECMs (two-way ANOVA with Tukey's post hoc test).

To investigate whether the ECM from HCjE-Gi cells differed with respect to time in culture, growth rates were determined for HCjE-Gi cells plated onto cell-derived ECMs from days 3, 5, 7, 14, 21, and 28 in comparison with assay-specific internal control wells on uncoated tissue culture wells treated with ammonium hydroxide to account for any assay-to-assay variability in cultures. Generally, the increases in cell density of those cells cultured on each ECM preparation followed a trend similar to that of the comparator cells cultured on TCP (Fig. 1A). However, the ECM-to-TCP cell number ratio was almost twofold higher on the day 3 ECM, 1 day after seeding, compared to other ECM preparations, which had ECM-to-TCP cell number ratios as follows: 1.94 ± 0.17 day 3 ECM, 0.86 ± 0.14 day 5 ECM, 0.91 ± 0.13 day 7 ECM, 0.41 ± 13 day 14, 0.88 ± 0.23 day 21 ECM. and 1.13 ± 0.40 day 28 ECM (*P* < 0.05 day 3 ECM vs. all other preparations, two-way ANOVA) (Fig. 1B). After this initial time point, ECM preparations were slightly superior to TCP in terms of supporting increased cell densities with the exception of day 28 ECM; however, none of the differences between the different ECM preparations reached statistical significance at an α of 0.05.

### Mass Spectrometry Identified Peptides of 101 Proteins in the ECM Secreted by HCjE-Gi Cells

**Figure 2.**
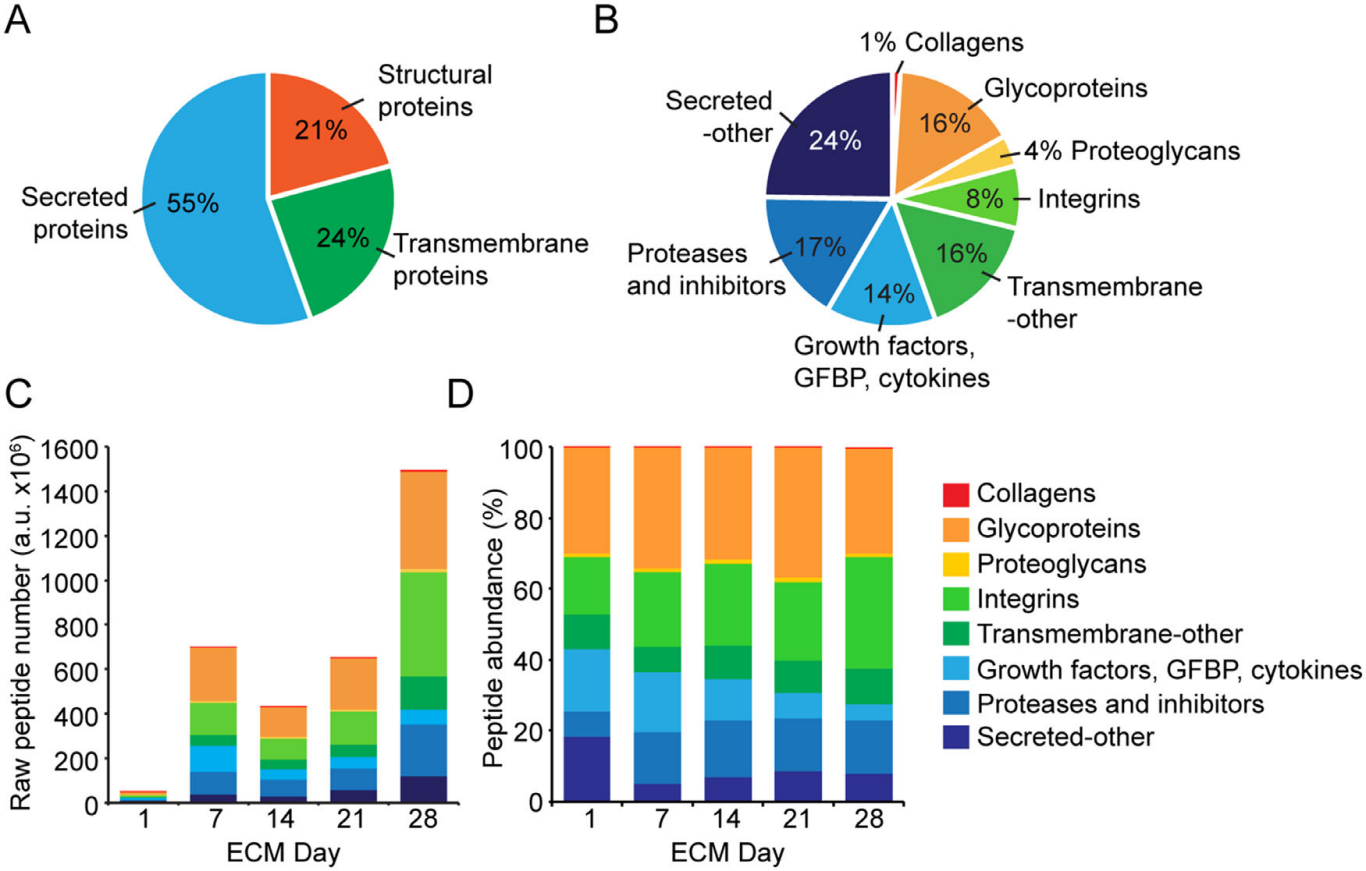
Mass spectrometry analysis of HCjE-Gi ECM preparations. (**A,** **B**) Breakdown of all peptides detected based on Unicode annotations. (**C**) Stacked column graph of raw peptide abundance. (**D**) Stacked column graph displayed as percentage of peptides detected based on Unicode annotation.

To identify potential reasons for the functional difference between ECMs, the composition of the ECMs was examined by mass spectrometry (Supplementary Data S1 file). Peptides from 928 proteins were detected, of which 101 were identified as either ECM or ECM-associated proteins using the Uniprot database. Of these 101 proteins, 21% were structural (collagens, glycoproteins, and proteoglycans), 24% transmembrane (integrins and other), and 55% secreted proteins, including GFs, GF binding proteins (GFBPs), cytokines, proteases and inhibitors, and other secreted proteins (Figs. 2A, 2B). As expected, the absolute number of ECM and ECM-associated protein peptides detected increased over time (Fig. 2C). However, as a proportion of the peptides detected at each time point, the integrins and the proteases and inhibitors all increased over the 42 days in culture: integrins, 16.2 ± 0.05% day 1 and 31.4 ± 1.2% day 42 (*P* < 0.001); proteases and inhibitors, 7.1 ± 0.6% day 1 and 15.1 ± 2.6% day 42 (*p* < 0.001) (Fig. 2D). The proportion of GFs, GFBPs, and cytokines decreased: 17.5 ± 3.1% day 1 and 4.4 ± 0.5% day 42 (*P* < 0.001) (Fig. 2D).

### HCjE-Gi Cells Produce a Predominantly LM332 Matrix

**Figure 3.**
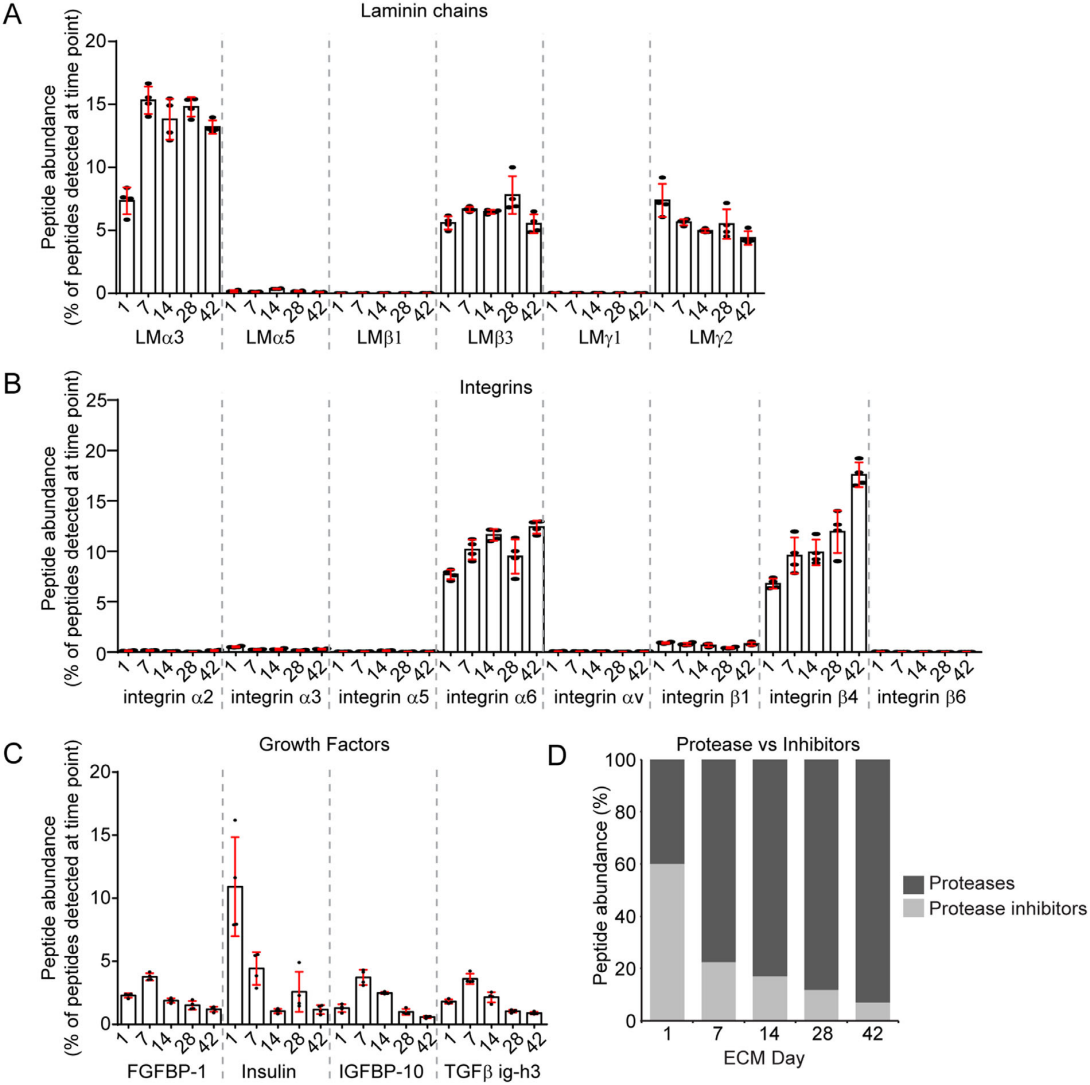
HCjE-Gi cells deposit a LM332-rich matrix; however, growth factor and protease abundance change over time in culture. The ECM preparations were processed by mass spectrometry. (**A–C**) The peptide abundance of the indicated protein is plotted as a proportion of all peptides detected at the indicated time point. Each dot represents an independent biological replicate, with bars representing mean and standard deviation error bars. (**D**) Stacked column graph of proteases and inhibitors as a ratio of all protease and inhibitor peptides detected at that time point. Values are plotted as the mean of three independent experiments; error bars are omitted for clarity.

The most abundant components—laminin (LM) α3, β3, and γ2 and the LM332 binding integrins α6 and β4—were highly represented at all time points (Supplementary Fig. S2), which is consistent with most epithelial cell types^31^^–^^33^ and also consistent with descriptive immunohistological findings from intact conjunctival tissue basement membranes,^34^^–^^36^ including LMα3, 7.3 ± 1.1% day 1 and 13.2 ± 0.5% day 42; LMβ3, 5.6 ± 0.5% day 1 and 5.5 ± 0.8% day 42; LMγ2, 7.4 ± 1.3% day 1 and 4.4 ± 0.5% day 42; integrin α6, 7.7 ± 0.5% day 1 and 12.4 ± 0.6% day 42; and integrin β4, 6.8 ± 0.5% day 1 and 17.6 ± 1.2% day 42 (Figs. 3A, 3B). The α5, β1, and γ1 laminin chains were all also detected, although they were each present at less than 1% of total peptides, as were all other integrins (Fig. 3A). Of the other laminin-binding proteins, syndecan 1 was more abundant than syndecan 4 at all of the time points, and thrombospondin 1 was consistently more abundant than thrombospondin 2 (Supplementary Fig. S2). Surprisingly, the archetypal basement membrane protein, collagen type IV, was not detected in this dataset nor were nidogens 1 or 2. Small amounts of collagen type XVIII (0.2 ± 0.03% day 1 and 0.3 ± 0.1% day 42) and perlecan (0.6 ± 0.1% day 1 and 0.4 ± 0.1% day 42) were detected (Supplementary Fig. S2).

### GFs, GFBPs, and Cytokines Decreased and Proteases Increased with Time

The absolute abundance of GFs, GFBPs, and cytokines as a proportion of the total peptides is less relevant due to their high activities at lower relative concentrations. Fourteen proteins were identified as GFs, GFBPs, and cytokines. Of these proteins, insulin was by far the most abundant on day 1 ECM (10.9 ± 3.9%), whereas at later time points insulin, fibroblast growth factor binding protein 1, insulin-like growth factor binding protein 10, and transforming growth factor β-induced protein ig-h3 were each present at similar levels, with a peak at day 7 (Fig. 3C, Supplementary Fig. S2). Consistent with a decrease in growth factor abundance, the proportion of proteases increased and the proportion of protease inhibitors decreased over the culture period (proteases, 39.9 ± 4.8% day 1 and 92.9 ± 1.7% day 42; inhibitors, 60.1 ± 4.8% day 1 and 7.1 ± 1.7% day 42) (Fig. 3D). Serine protease high-temperature requirement A1 was the most abundant and displayed a steady increase over the time course (2.2 ± 0.2% day 1 and 13.3 ± 2.6% day 42) (Supplementary Fig. S2).

### Fibronectin and α-2-HS-GP Are Enriched in Early ECM Relative to Late ECM Preparations

**Figure 4.**
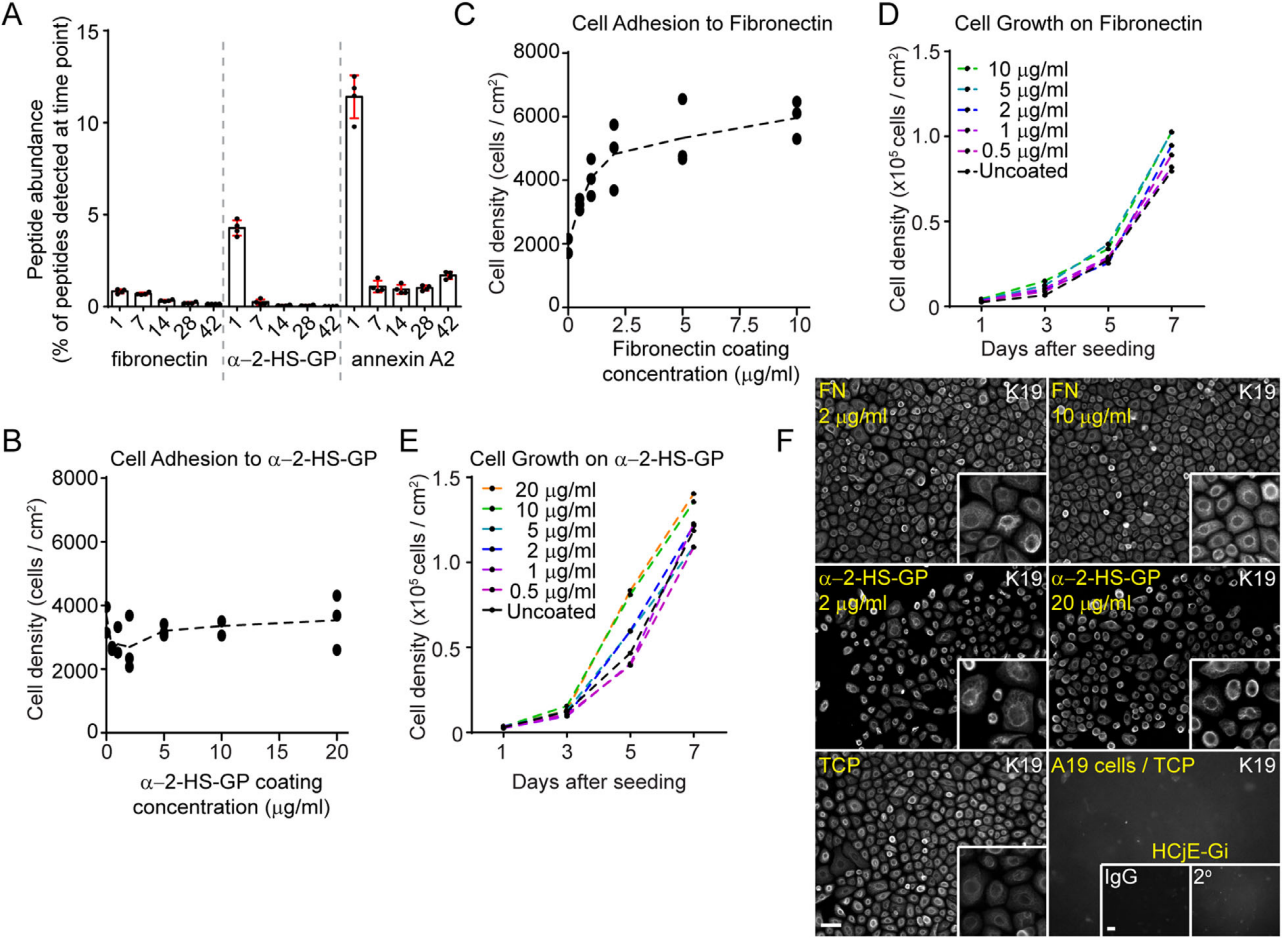
Fibronectin and α-2-HS-GP are enriched in early ECMs and support HCjE-Gi cell adhesion and growth. (**A**) The peptide abundance of fibronectin, α-2-HS-GP, and annexin A2 is plotted as a percentage of peptides detected at the indicated time points. (**B**) Density of HCjE-Gi cells measured 3 hours after seeding onto indicated concentrations of fibronectin. (**C**) Growth curves of HCjE-Gi cells plated onto different fibronectin concentrations. (**D**) Density of HCjE-Gi cells measured 3 hours after seeding onto indicated concentrations of α-2-HS-GP. (**E**) growth curves of HCjE-Gi cells plated onto different α-2-HS-GP concentrations. (**F**) HCjE-Gi cells plated onto TCP coated with indicated concentrations of fibronectin or α-2-HS-GP or left uncoated (TCP) were processed for indirect immunofluorescence microscopy on culture day 7 with monoclonal antibodies against keratin 19. HCjE-Gi cells on TCP were also processed with rabbit IgG or secondary antibodies as negative controls. Retinal pigment epithelial cells (ARPE-19) were processed in the same way as a negative control for antibodies specificity. *Scale bars*: 50 µm.

Analysis of known adhesive proteins within our preparations revealed that, whereas vitronectin and nephronectin were barely detected, fibronectin and α-2-HS-GP were present at 0.8 ± 0.1% and 4.3 ± 0.4%, respectively, in day 1 ECM (Fig. 4A, Supplementary Fig. S1). Importantly, both of these proteins were reduced dramatically by proportion of total peptides in the later ECM preparations. Interestingly, the highest proportion of annexin A2, known to interact with α-2-HS-GP and alter its functions,^37^ was also found in day 1 ECM (11.4 ± 1.2%) and was dramatically reduced by day 7 (1.1 ± 0.3%) (Fig. 4A). By absolute abundance, α-2-HS-GP peaked at day 1 (2.08 × 10^6^ ± 0.39 × 10^6^ arbitrary units), and fibronectin peaked at day 7 (4.92 × 10^6^ ± 1.05 × 10^6^ arbitrary units). These data highlighted these two proteins as warranting further investigation.

### Fibronectin Supported HCjE-Gi Cell Adhesion and Expansion

Fibronectin has long been associated with cell adhesion and growth of epithelial cells,^32^^,^^38^^–^^41^ and its detection in our hypothesis-independent screen gave us confidence in our approach. To confirm that provision of a fibronectin at time of plating improved the adhesion and expansion of HCjE-Gi cultures and for comparison with α-2-HS-GP coating, we coated TCP with 0.5- to 10-µg/mL fibronectin solutions and assessed the number of cells adhered after 3 hours and growth curves over 7 days compared with uncoated TCP. These assays revealed that, as expected, as fibronectin coating concentrations increased up to a plateau of 5 µg/mL, HCjE-Gi cell adhesion increased by around threefold compared with TCP (2000 ± 260 cells/cm^2^ on TCP and 5300 ± 1100 cells/cm^2^ on 5-µg/mL fibronectin; *P* < 0.001) (Fig. 4B). The cell population densities increased on all fibronectin coating concentrations compared with controls, with 1.26-fold higher cell numbers on culture day 7 on 5- to 10-µg/mL fibronectin compared to TCP (81,000 ± 22,000 cells/cm^2^ on TCP; 102,000 ± 8000 cells/cm^2^ on 5-µg/mL fibronectin; and 102,000 ± 10,000 cells/cm^2^ on 10-µg/mL fibronectin) (*P* < 0.05; two-way ANOVA).

### α-2-HS-Glycoprotein Increased HCjE-Gi Cell Growth but Not Cell Adhesion

Coating of culture plates with α-2-HS-GP from 0.5 to 20 µg/mL did not support increased cell adhesion compared with TCP (3700 ± 500 cells/cm^2^ on TCP vs. 3500 ± 900 cells/cm^2^ on 20-µg/mL α-2-HS-GP; *P* > 0.05) (Fig. 4D); however, this coating did increase the rate at which cell density increased in an approximately dose-dependent manner (Fig. 4E). Specifically, when cultured on α-2-HS-GP pre-adsorbed from 10 to 20 µg/mL, there were 1.14 to 1.18 times more cell numbers observed compared with TCP on culture day 7 (119,000 ± 12,000 cells/cm^2^ on TCP; 136,000 ± 8000 cells/cm^2^ on 10-µg/mL α-2-HS-GP; and 140,000 ± 6000 cells/cm^2^ on 20-µg/mL α-2-HS-GP) (*P* *<* 0.01; two-way ANOVA). Processing cells for indirect immunofluorescence microscopy with the antibodies against conjunctival protein keratin 19, simple epithelial keratins 7, 8, and 18; corneal keratin 3; and stratified epidermal keratin 1 revealed that culturing HCjE-Gi cells on either fibronectin or α-2-HS-GP did not induce de- or transdifferentiation (Fig. 4F, Supplementary Fig. S3).

## Discussion

In this study, we have shown how the composition of the ECM secreted by HCjE-Gi cells changes over time in culture. The ECM deposited after short periods of culture is superior at supporting the attachment and growth of conjunctival epithelial cells compared with that deposited at later time points. Consistent with this, mass spectrometry revealed that early ECM consisted of higher proportions of adhesive proteins including fibronectin and α-2-HS-GP, the latter of which has not been studied in the conjunctiva before. When provided as a substrate coating at the time of plating, both of these proteins improved the culture of HCjE-Gi cells; fibronectin, a known adhesive protein, as expected increased cell adhesion, and both fibronectin and α-2-HS-GP increased the rate at which cell numbers increased in a dose-dependent manner. Importantly, these data reveal α-2-HS-GP as a new component that could aid the ex vivo culture of conjunctival epithelial cells.

α-2-HS-GP is primarily produced by hepatocytes in adults, where its production is regulated by systemic inflammation.^42^^,^^43^ Indeed, as there are no reports of its expression in the conjunctiva or in the ocular surface, it is unlikely we would have selected it as a candidate protein had we not adopted a hypothesis-independent approach. However, studies on intestinal epithelial cells and breast carcinoma cells have shown that α-2-HS-GP does have adhesive properties, which are mediated via annexins A2 and A6, respectively.^37^ α-2-HS-GP has also been shown to increase cell migration and wound closure rates of epidermal keratinocyte to a level similar to that when KSF is supplemented with bovine pituitary extract and epidermal growth factor.^44^^,^^45^ α-2-HS-GP is the human homolog of fetuin A, which is found in fetal calf serum, one of the many cell-adhesive proteins present in serum used in tissue culture.^42^^,^^44^ In our studies, the peptide identification (P02765) was identified as that of human origin on the Uniprot database, thus clarifying that the protein identified was not obtained from growth supplements used in the culture media but rather came from cells synthesizing and depositing their own protein into the ECM.

It is important to stress that our findings do not necessarily imply that α-2-HS-GP is acting alone. Throughout the time course, and as expected, LM332 remained the most abundant matrix protein detected. As the cells plated onto α-2-HS-GP or fibronectin continue to express and deposit LM332, almost certainly synergistic effects between these proteins are driving the change in cell behavior. Whether they each act upon the same pathway or through distinct mechanisms is as yet unknown but could represent a valuable avenue for future research.

Although we have focused on adhesive proteins that could ultimately be used for substrate modification, all of the other data produced by the mass spectrometry experiments represent a resource that contributes to the knowledge on conjunctival ECMs and could be exploited to improve culture conditions. The data specifically revealed additional interesting patterns of expression of GF-related proteins, with the highest proportion of GFs and their binding proteins being observed in the early ECM samples. Moreover, alongside the reduction in ECM-associated GFs in later preparations, we observed an increase in proteases. The proteases may cleave the GFs from the other ECM proteins, and we cannot rule out removal of the GFs during sample preparation; however, assuming that the reduction represents decreased production or increased turnover, these data raise the possibility that supplementing longer term conjunctival cell cultures with the GFs that are relatively depleted over time may help continue cultures.

We chose to focus our studies on the widely used HCjE-Gi cell line,^46^^,^^47^ as we required large cell numbers and tight controls between replicates to reduce heterogeneity. Extending these studies into primary cultures and a wider set of culture conditions is now a priority. Moreover, although we have investigated α-2-HS-GP in isolation, it is likely that a combination of substrate and culture modifications can be identified that will further improve culture conditions. Indeed, the aim of conjunctival cell transplantation is to produce a functional tissue construct that is also able to maintain goblet cells and produce mucins required for the health of the ocular surface. Future studies are required, therefore, to assess the growth and differentiation of goblet cells and types of mucin production. We hope that the provision of this mass spectrometry dataset to the field will generate hypotheses that can be used for future such studies.

It should be noted that, in this study, we used NH_4_OH, a hypertonic solution that disrupts the cells through osmotic shock,^48^ to generate our ECM extracts. Alkaline solutions denature proteins, solubilize cell organelles, and remove nucleic acids^48^; however, the NH_4_OH protocol is also known to isolate the transmembrane proteins that are associated with the ECM proteins.^25^ Consistent with this, we were able to confirm a high abundance of α6β4 integrin in our samples. A potential drawback of using alkaline solutions is that they have been shown to be capable of removing glycosaminoglycans from collagenous tissues.^49^ Therefore, the panel of ECM proteins we have identified may be an incomplete measure of those deposited by conjunctival cells. We selected to accept this potential limitation, as the functional studies were performed on matrix prepared in the same way.

## Conclusions

Together, the data presented here have revealed a new candidate protein that could be used to enhance the growth of conjunctival epithelial cells ex vivo and which could now be tested for its suitability to support conjunctival cell transplantation. Understanding the roles of this protein with regard to the growth of conjunctival epithelial and goblet cells can help the development of specific peptides from the larger protein molecules that could be used in the surface modification of synthetic substrates.

## Supplementary Material

Supplement 1

Supplement 2

Supplement 3

Supplement 4

Supplement 5
