# Supplementary material for: Mass Spectrometry Reveals α-2-HS-Glycoprotein as a Key Early Extracellular Matrix Protein for Conjunctival Cells
Source: Invest Ophthalmol Vis Sci. 2020 Mar 30;61(3):44. doi: 10.1167/iovs.61.3.44 (PMC7401837; doi:10.1167/iovs.61.3.44)
Supplement: Supplement 1 [file iovs-61-3-44_s001.pdf]

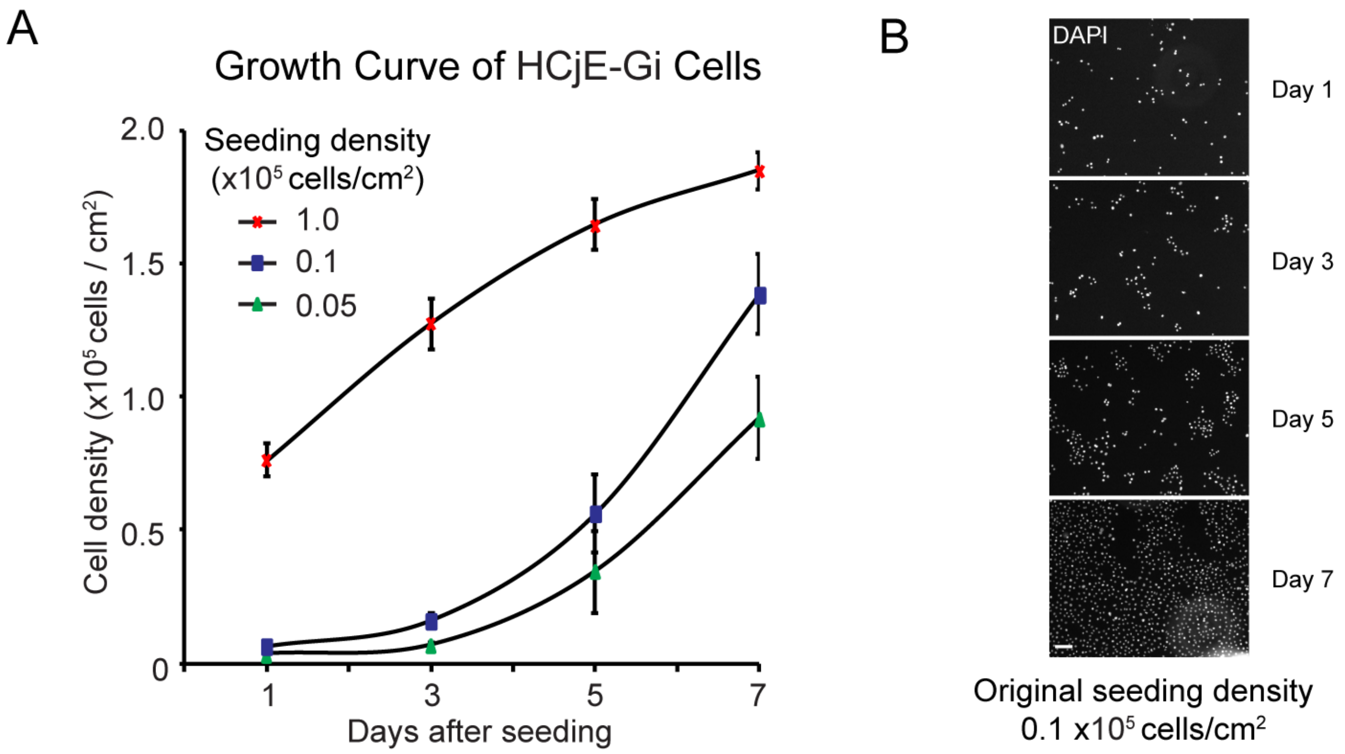

Supplemental Figure 1. hCjE-Gi growth conditions. A. HCjE-Gi cells were plated at 1, 0.1 or 0.05 x 10^5 cells/cm^2 on 48 well plates. 1, 3, 5 and 7 days after plating, plates were fixed stained with DAPI and cell density calculated. Points represent mean ± SD from 3 biological repeats. B. Representative DAPI images from HCjE-Gi plated at 0.1 $x10^5$ cells/cm^2. Scale bar 50 μm.
