# Supplementary material for: Mass Spectrometry Reveals α-2-HS-Glycoprotein as a Key Early Extracellular Matrix Protein for Conjunctival Cells
Source: Invest Ophthalmol Vis Sci. 2020 Mar 30;61(3):44. doi: 10.1167/iovs.61.3.44 (PMC7401837; doi:10.1167/iovs.61.3.44)
Supplement: Supplement 2 [file iovs-61-3-44_s002.pdf]

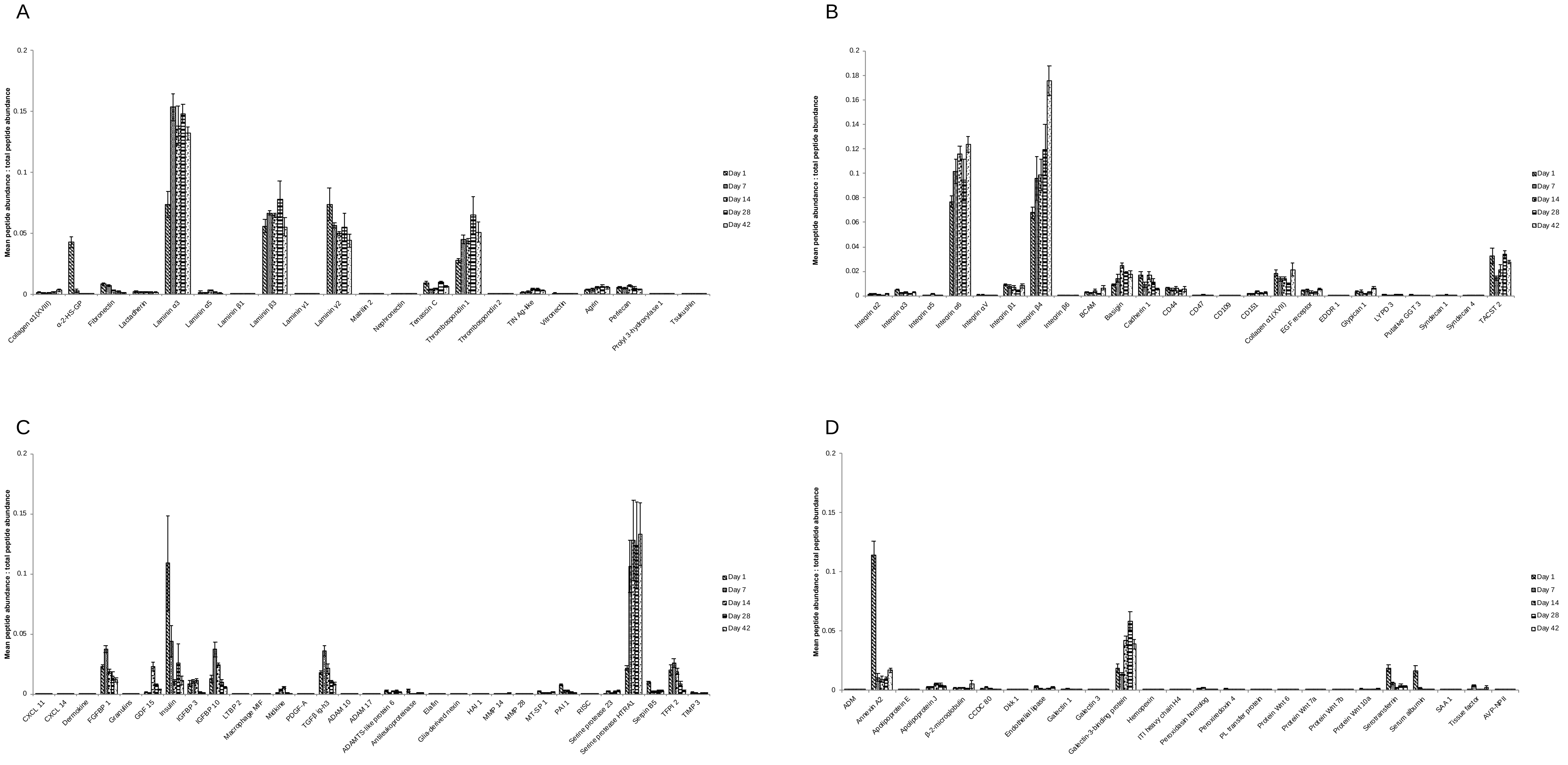

Supplemental Figure 2: Proportions of structural proteins (A), transmembrane proteins (B) and secreted proteins (C and D) in the extracellular matrix secreted by HCjE-Gi cells from days 1 to 42. Data are presented as mean peptide abundance: total peptide abundance ± SD, n = 4.
