# Supplementary material for: Mass Spectrometry Reveals α-2-HS-Glycoprotein as a Key Early Extracellular Matrix Protein for Conjunctival Cells
Source: Invest Ophthalmol Vis Sci. 2020 Mar 30;61(3):44. doi: 10.1167/iovs.61.3.44 (PMC7401837; doi:10.1167/iovs.61.3.44)
Supplement: Supplement 3 [file iovs-61-3-44_s003.pdf]

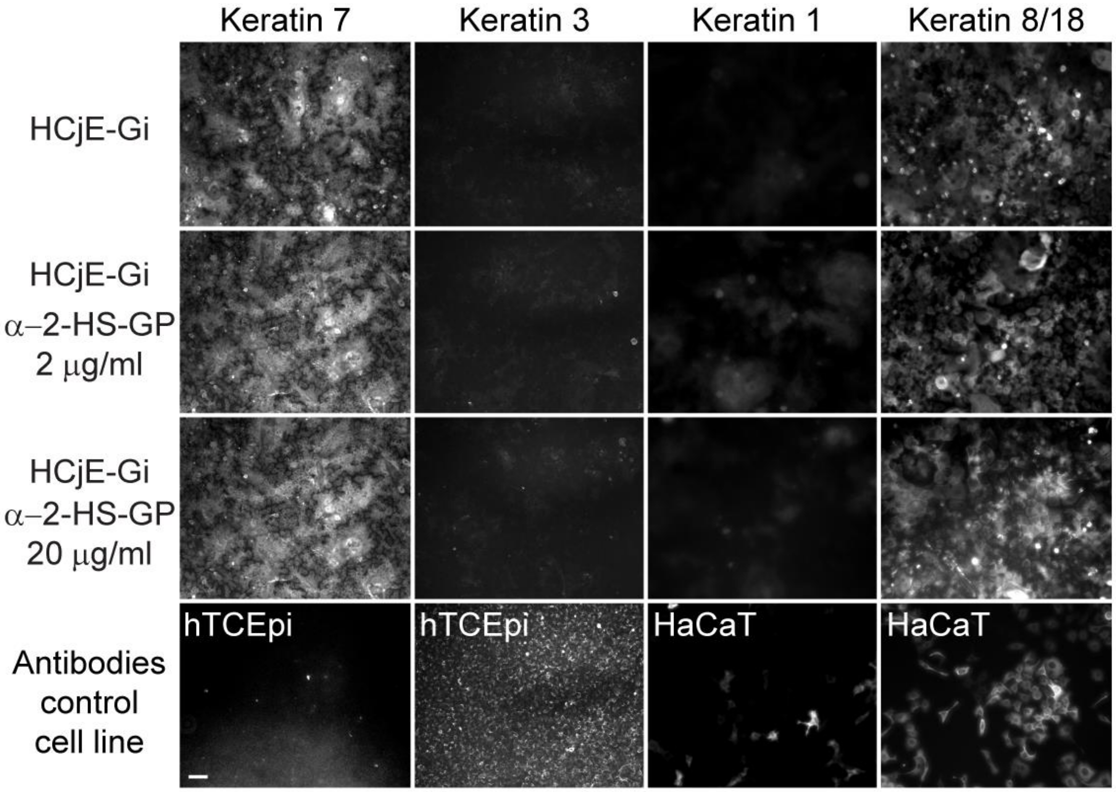

Supplemental Figure 3. HCjE-Gi retain conjunctival marker expression when cultured on α–2-HS-HP. HCjE-Gi cells plated onto tissue culture polystyrene (top panels) or TCP coated with indicated concentrations of α2-HS-GP were processed for indirect immunofluorescence microscopy on culture day 7 with antibodies against keratin 7, 3, 1 or 8 and 18 as indicated. Corneal epithelial cells (hTCEpi) or epidermal keratinocytes (HaCaT) were processed in the same way as controls for antibodies specificity. Scale bars 100 μm.
