# Supplementary material for: Mass Spectrometry Reveals α-2-HS-Glycoprotein as a Key Early Extracellular Matrix Protein for Conjunctival Cells
Source: Invest Ophthalmol Vis Sci. 2020 Mar 30;61(3):44. doi: 10.1167/iovs.61.3.44 (PMC7401837; doi:10.1167/iovs.61.3.44)
Supplement: Supplement 4 [file iovs-61-3-44_s004.pdf]

Supplementary methods

LC-MS/MS Instrumentation and software

Data-dependent LC-MS/MS analyses were conducted on a QExactiveHF quadrupole-Orbitrap mass spectrometer coupled to a Dionex Ultimate 3000 RSLC nano-liquid chromatograph (*Hemel Hempstead, United Kingdom*). Sample digests (15 µL) was loaded onto a trapping column (Acclaim PepMap 100 C18, 75 µm x 2 cm, 3 µm packing material, 100Å) using a loading buffer of 0.1 % TFA (v/v) and 2 % acetonitrile (v/v) (*Fluka, Munich, Germany, 34668*) in water for seven minutes at a flow rate of 12 µL/minute. The trapping column was then set in-line with an analytical column (*EASY-Spray PepMap RSL C18, ThermoFisher, Waltham, United States,* 75 µm x 50 cm, 2 µm packing material, 100Å) and the peptides eluted using a linear gradient of 96.2 % A (0.1% formic acid (v/v) (*Biosolve 0006914143BS*)), 3.8% B (0.1% formic acid (v/v) in water : acetonitrile [80:20] (v/v)) to 50 % A : 50 % B over thirty minutes at a flow rate of 300 nL/minute, followed by washing with 1 % A : 99 % B for five minutes and re-equilibration of the column to starting conditions. The column was maintained at 40 °C, and the effluent introduced directly into the integrated nano-electrospray ionisation source operating in positive ion mode. The mass spectrometer was operated in data dependent acquisition mode with survey scans between mass/charge ratios 350-2000 acquired at a mass resolution of 60,000 (full width half maximum) at mass/charge ratio 200. The maximum injection time was 100 milliseconds, and the automatic gain control was set to 3×10^6. The eighteen most intense precursor ions with charge states of between 2^+ and 5^+ were selected for MS/MS with an isolation window of 1.2 m/z units. The maximum injection time was 45 milliseconds, and the automatic gain control was set to 1×10^5. Fragmentation of the peptides was by higher-energy collisional dissociation using a stepped normalised collision energy of 28 – 30 %. Dynamic exclusion of mass/charge ratio values to prevent repeated fragmentation of the same peptide was used with an exclusion time of 20 seconds.

Raw mass spectral data files were processed using Progenesis-QI (*version 2; Nonlinear Dynamics*) to determine the total protein abundances. Protein quantification was based on averaging the individual abundances for every unique peptide for each protein and comparing them relatively across sample runs and between sample groups. The raw data file was imported into Progenesis QI for Proteomics (*version 3.0.5995.47167 Nonlinear Dynamics, Newcastle upon Tyne United Kingdom; Waters Company*). Samples were aligned according to

retention time using a combination of manual and automatic alignment. Peak picking parameters were applied with sensitivity set to maximum and features with charges of 2^+ to 7^+ were retained. A Mascot Generic File, created by Progenesis, was searched against the human reviewed database from Uniprot (20 187 sequences). Trypsin was specified as the protease with one missed cleavage allowed and with fixed carbamidomethyl modification for cysteine and variable oxidation modification for methionine. A precursor mass tolerance of 10 parts per million and a fragment ion mass tolerance of 0.01 Da were applied. The results were then filtered to obtain a peptide false discovery rate of 1 %.
